# Anti-Inflammatory Effect of Atorvastatin and Rosuvastatin on Monosodium Urate-Induced Inflammation through IL-37/Smad3-Complex Activation in an In Vitro Study Using THP-1 Macrophages

**DOI:** 10.3390/ph17070883

**Published:** 2024-07-03

**Authors:** Seong-Kyu Kim, Jung-Yoon Choe, Ji-Won Kim, Ki-Yeun Park, Boyoung Kim

**Affiliations:** 1 Division of Rheumatology, Department of Internal Medicine, Catholic University of Daegu School of Medicine, Daegu 42472, Republic of Korea; 2 Arthritis and Autoimmunity Research Center, Catholic University of Daegu, Daegu 42472, Republic of Korea

**Keywords:** monosodium urate, interleukin-37, statin, Smad3, caspase-1

## Abstract

**Objective**: The pleiotropic effect of hydroxy-3-methylglutaryl coenzyme A (HMG-CoA) reductase inhibitors (statins) is responsible for potent defense against inflammatory response. This study evaluated the inhibitory effects of HMG-CoA reductase inhibitors on the monosodium urate (MSU)-induced inflammatory response through the regulation of interleukin-37 (IL-37) expression. **Methods**: Serum was collected from patients with gout (*n* = 40) and from healthy controls (*n* = 30). The mRNA and protein expression of the target molecules IL-1β, IL-37, caspase-1, and Smad3 were measured in THP-1 macrophages stimulated with MSU, atorvastatin, or rosuvastatin using a real-time quantitative polymerase chain reaction and Western blot assay. Transfection with IL-1β or Smad3 siRNA in THP-1 macrophages was used to verify the pharmaceutical effect of statins in uric-acid-induced inflammation. **Results**: Serum IL-37 levels in gout patients were significantly higher than in controls (*p* < 0.001) and was associated with the serum uric acid level (*r* = 0.382, *p* = 0.008). THP-1 cells stimulated with MSU markedly induced IL-37 mRNA expression and the transition of IL-37 from the cytoplasm to the nucleus. Recombinant IL-37 treatment dose-dependently inhibited activation of caspase-1 and IL-1β in MSU-induced inflammation. Atorvastatin and rosuvastatin attenuated caspase-1 activation and mature IL-1β expression but augmented translocation of IL-37 from the cytoplasm to the nucleus. Atorvastatin and rosuvastatin induced phosphorylation of Smad3 in THP-1 cells treated with MSU crystals. Statins potently attenuated translocation of IL-37 from the cytoplasm to the nucleus in THP-1 macrophages transfected with Smad3 siRNA compared to cells with negative control siRNA. **Conclusions**: This study revealed that statins inhibit the MSU-induced inflammatory response through phosphorylated Smad3-mediated IL-37 expression in THP-1 macrophages.

## 1. Introduction

Gout is a crystal-induced inflammatory disease triggered by excessive deposition of monosodium urate (MSU) in intraarticular and periarticular structures and other body organs [1,2]. Recently, it has been recognized that the innate immune system mediated through activation of NLRP3 inflammasome triggered by MSU crystals is involved in the pathogenesis of gouty arthritis [3,4]. The NLRP3 inflammasome is an intracellular multi-protein signaling platform that leads to proteolytic cleavage of pro-interleukin-1β (pro-IL-1β) into mature IL-1β, a proinflammatory cytokine mainly responsible for uric-acid-induced inflammation. Various molecules negatively regulate NLRP3 inflammasome activation such as A20, nitric oxide, carbon monoxide, and the aryl hydrocarbon receptor [5,6], suggesting that identification of novel negative regulators of NLRP3 inflammasome activation might offer therapeutic options for control of uric-acid-induced inflammation. 

IL-37, one member of the IL-1 cytokine family, is a potent anti-inflammatory cytokine that primarily reduces inflammatory response by suppressing production of proinflammatory cytokines and attenuating the expression of transcriptional cytokines [7,8,9,10]. IL-37 is up-regulated by inflammatory stimuli including various proinflammatory cytokines, TLR ligands, or lipopolysaccharide via diverse signal transduction pathway. Clinically, IL-37 plays an important role in the mechanisms that protect against immune/inflammatory responses in chronic inflammatory and/or autoimmune diseases and against the progression and development of diverse tumors. Anti-inflammatory effects of IL-37 have been extensively identified in diverse autoimmune and inflammatory rheumatic diseases such as rheumatoid arthritis (RA) [11,12], systemic lupus erythematosus (SLE) [13], osteoarthritis (OA) [14], and ankylosing spondylitis (AS) [15] through suppression of proinflammatory cytokines or inflammatory cells. Consistently, IL-37 expression in serum and peripheral blood mononuclear cells (PBMCs) in gout patients was also significantly higher than in healthy controls [16,17]. In addition, other experimental studies have shown that IL-37 exerted a potent anti-inflammatory effect by reducing the release of proinflammatory cytokines in uric-acid-induced inflammation [18,19]. 

Hydroxy-3-methylglutaryl coenzyme A (HMG-CoA) reductase inhibitors, generally referred to as “statins”, are inhibitors of cholesterol biosynthesis [20,21,22]. Accumulating evidence also proposes that HMG-CoA reductase inhibitors have some favorable cholesterol-independent or pleiotropic properties such as the improvement of endothelial dysfunction, abnormal glucose metabolism, and inflammation. IL-1β is a critical proinflammatory cytokine that is responsible for the development of uric-acid-induced sterile inflammatory diseases like gout [3,4]. Some studies demonstrated that variable HMG-CoA reductase inhibitors markedly suppressed mature IL-1β release, generated during an MSU-induced inflammatory response [23,24]. Data on whether HMG-CoA reductase inhibitors affect the immunomodulatory effect of IL-37 are insufficient, although some studies demonstrated that IL-37 could be implicated in the pathogenesis of uric-acid-induced inflammation [16,17,18,19]. There is a lack of data on the relationship between HMG-CoA reductase inhibitors and IL-37 expression in the pathogenesis of gout. Thus, this study investigated whether the therapeutic effect of HMG-CoA reductase inhibitors is related to the regulation of IL-37 expression and its activation in uric-acid-induced inflammation using THP-1 macrophages.

## 2. Results

### 2.1. IL-37 Expression in MSU-Induced Inflammation

**Figure 1:**
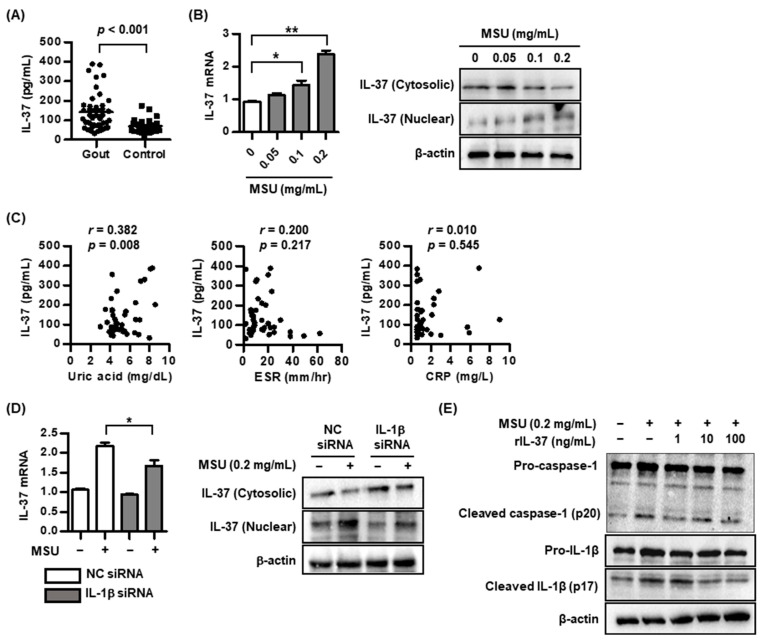
IL-37 expression in gout and uric-acid-induced inflammation. (**A**) Comparison of serum IL-37 level between gout patients (*n* = 40) and controls (*n* = 30). (**B**) Expression of IL-37 mRNA and IL-37 protein in cytoplasm and nucleus in THP-1 macrophages treated with MSU crystals. (**C**) Correlation of IL-37 level with uric acid, ESR, and CRP in serum in gout patients. (**D**) Expression of IL-37 mRNA and IL-37 protein in cytoplasm and nucleus in THP-1 macrophages transfected with IL-1β siRNA under stimulation with MSU crystals. (**E**) Expression of active caspase-1 and IL-1β in THP-1 macrophages stimulated with recombinant IL-37. * *p* < 0.05 and ** *p* < 0.01. Values presented as mean ± SEM of three independent experiments. The representative images are illustrated after three independent experiments. Abbreviation: MSU, monosodium urate; IL-37, interleukin-37; IL-1β, interleukin-1β; ESR, erythrocyte sedimentation rate; CRP, C-reactive protein.

We compared serum IL-37 levels between gout patients (*n* = 40, 63.0 ± 13.8 years) and controls (*n* = 30, 58.4 ± 12.0 years), measured by the ELISA method. Gout patients showed a higher serum IL-37 level, compared to controls (*p* < 0.001) (Figure 1A). We assessed whether MSU crystals can stimulate IL-37 expression in THP-1 macrophages. Stimulation with MSU at concentrations of 0.1 and 0.2 mg/mL in THP-1 macrophages gradually induced IL-37 mRNA expression in a dose-dependent manner (* *p* < 0.05 and ** *p* < 0.01 compared to unstimulated cells, respectively) (Figure 1B). Consistently, IL-37 protein expression in the cytoplasm of THP-1 macrophages treated with MSU was reduced in a dose-dependent manner, whereas nuclear IL-37 expression was gradually increased. Next, we evaluated whether serum uric acid levels and acute-phase reactants are related to serum IL-37 levels in gout patients. A positive correlation was noted between serum IL-37 level and serum uric acid in patients with gout (*r* = 0.382, *p* = 0.008) (Figure 1C). However, acute-phase reactants including erythrocyte sedimentation rate (ESR, mm/h) and C-reactive protein (CRP, mg/L) in gout patients were not related to serum IL-37 levels (*p* > 0.05 of both). Considering that IL-37 has been identified as a potential anti-inflammatory cytokine against proinflammatory cytokine IL-1β generated in uric-acid-induced inflammation, THP-1 macrophages transfected with IL-1β siRNA significantly suppressed IL-37 mRNA expression compared to cells with NC siRNA (Figure 1D). In addition, to verify the role of IL-37 in gout, THP-1 macrophages stimulated with recombinant IL-37 dose-dependently suppressed the activation of caspase-1 and IL-1β in MSU-induced inflammation (Figure 1E). This suggests that IL-37 expression could be tightly regulated by IL-1β in uric-acid-induced inflammation. 

### 2.2. Effect of Statins on Caspase-1, IL-1β, and IL-37 Expression in the MSU-Induced Inflammatory Response 

**Figure 2:**
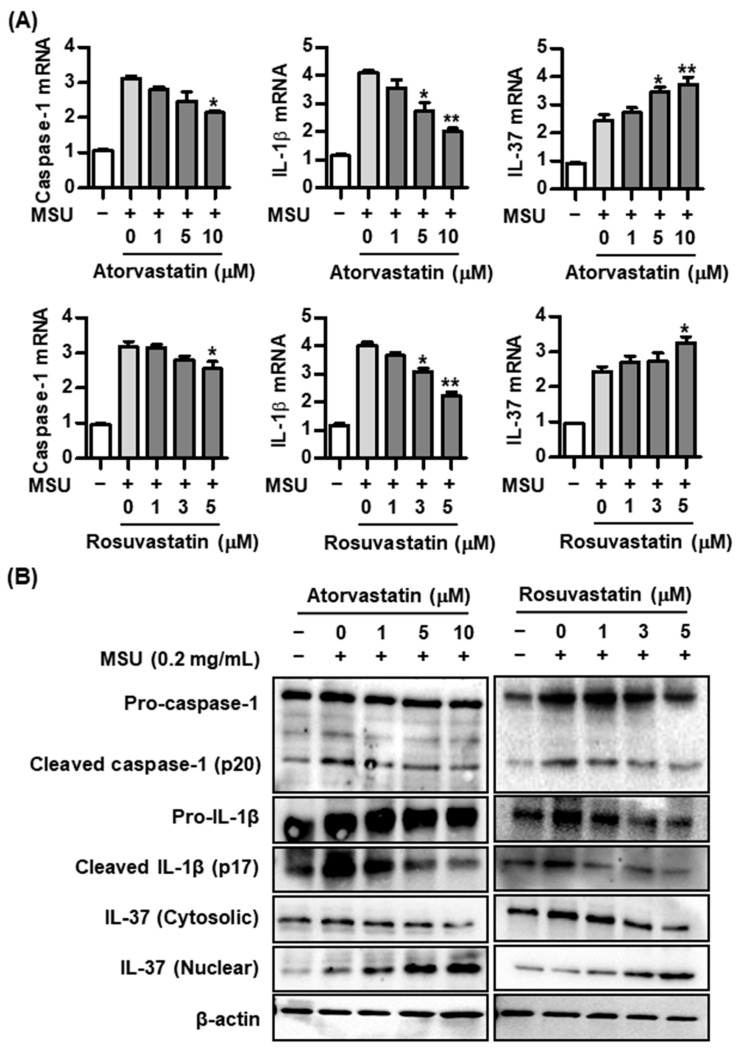
Changes in caspase-1, IL-1β, and IL-37 mRNA and protein expression by atorvastatin or rosuvastatin in THP-1 macrophages treated with MSU crystals. (**A**) Expression of IL-37 mRNA after addition of atorvastatin or rosuvastatin in THP-1 macrophages treated with MSU crystals. (**B**) Expression of cleaved caspase-1, cleaved IL-1β, and IL-37 protein in cytoplasm and nucleus after addition of atorvastatin or rosuvastatin in THP-1 macrophages treated with MSU crystals. * *p* < 0.05 and ** *p* < 0.01. Values presented as mean ± SEM of three independent experiments. The representative images are illustrated after three independent experiments. Abbreviation: MSU, monosodium urate; IL-37, interleukin-37; IL-1β, interleukin-1β.

We evaluated the effects of HMG-CoA reductase inhibitors including atorvastatin and rosuvastatin on caspase-1, IL-1β, and IL-37 expression in THP-1 macrophages treated with 0.2 mg/mL of MSU crystals. Atorvastatin suppressed caspase-1 and IL-1β mRNA expression at doses of 5.0 and/or 10.0 μM but increased IL-37 mRNA expression in THP-1 macrophages treated with MSU at the same dosages (* *p* < 0.05 and ** *p* < 0.01, compared to cells without atorvastatin treatment) (Figure 2A). Similarly, rosuvastatin at a dose of 3.0 or 5.0 μM in uric-acid-induced inflammation inhibited caspase-1 and IL-1β mRNA expression, whereas IL-37 mRNA expression in THP-1 cells stimulated with MSU was increased by rosuvastatin at a dose of 5.0 μM (* *p* < 0.05 and ** *p* < 0.01, compared to cells without rosuvastatin treatment) (Figure 2A).

Consistently, increased the expression of caspase-1 and IL-1β proteins in THP-1 macrophages stimulated with MSU alone was dose-dependently attenuated in cells treated with atorvastatin at 1.0, 5.0, or 10.0 μM (Figure 2B). Atorvastatin treatment markedly increased IL-37 expression in the nucleus of cells stimulated with MSU crystals compared to cells stimulated with MSU alone. However, IL-37 expression in the cytoplasm was dose-dependently reduced by atorvastatin. In addition, rosuvastatin treatment significantly suppressed the activation of caspase-1 and IL-1β protein from the inactive forms of these proteins in a dose-dependent manner (Figure 2B). Translocation of IL-37 from the cytoplasm into the nucleus was augmented by treatment with rosuvastatin under stimulation of MSU.

### 2.3. Effects of Statins on Interactions with Smad3 and IL-37 in MSU-Induced Inflammation

**Figure 3:**
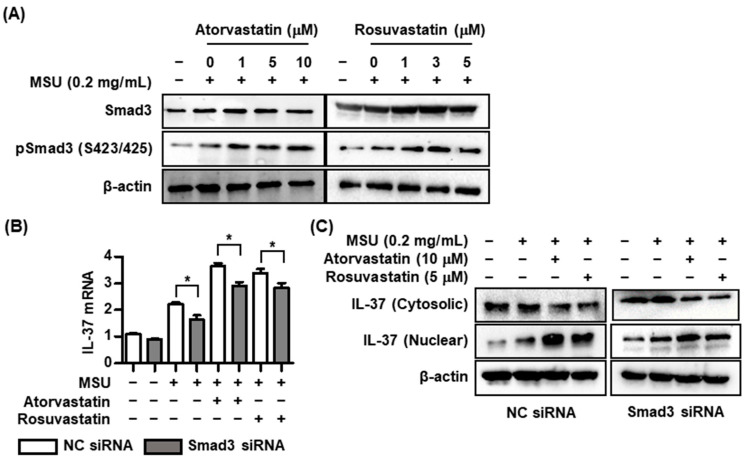
Changes in Smad3 and IL-37 expression by atorvastatin or rosuvastatin in MSU-induced inflammation. (**A**) Expression of phosphorylation of Smad3 after addition of atorvastatin or rosuvastatin in THP-1 macrophages treated with MSU crystals. (**B**) Comparison of IL-37 mRNA expression between cells transfected with NC siRNA and Smad3 siRNA under stimulation with MSU crystals. (**C**) Comparison of IL-37 protein expression in cytoplasm and nucleus between cells transfected with NC siRNA and Smad3 siRNA under stimulation with MSU crystals. * *p* < 0.05. Values presented as mean ± SEM of three independent experiments. The representative images are illustrated after three independent experiments. Abbreviation: MSU, monosodium urate; IL-37, interleukin-37.

Mature IL-37 binds to phosphorylated Smad3 to lead to the generation of an IL-37/Smad3 complex in the cytoplasm that then moves into the nucleus [7,8,9,10]. Finally, IL-37 binding to Smad3 results in up- or down-regulation of diverse proinflammatory or anti-inflammatory gene expression [10]. Here, it was determined whether HMG-CoA reductase inhibitors regulated phosphorylation of Smad3 in the condition of MSU-crystal-induced stimulation in THP-1 macrophages. We found that atorvastatin and rosuvastatin significantly induced the phosphorylation of Smad3 in a dose-dependent manner (Figure 3A). This suggests that HMG-CoA reductase inhibitors augment the generation of the IL-37/Smad3 complex in uric-acid-induced inflammation. In the assessment of the effect of statins on Smad3-dependent IL-37 expression, IL-37 mRNA expression stimulated by atorvastatin and rosuvastatin in cells transfected with Smad3 siRNA was down-regulated, rather than in those with NC siRNA (Figure 3B). Cells transfected with Smad3 siRNA under stimulation with MSU alone showed lower IL-37 mRNA expression than did those with NC siRNA. In addition, stimulation with atorvastatin or rosuvastatin markedly decreased the translocation of IL-37 from the cytoplasm to the nucleus in THP-1 macrophages transfected with Smad3 siRNA under stimulation with MSU (Figure 3C). 

### 2.4. Effect of Statins on Proteolytic Activity in the Process of IL-37 Activation

**Figure 4:**
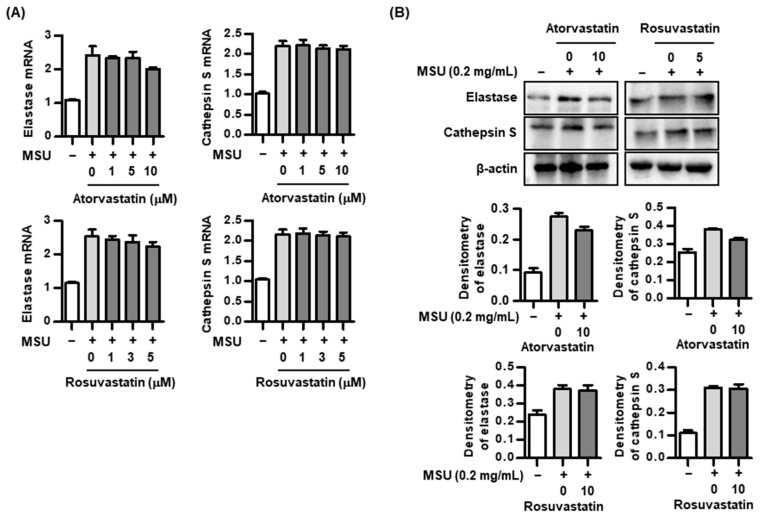
Changes in proteolytic enzymes by atorvastatin or rosuvastatin in THP-1 macrophages. (**A**,**B**) Expression of elastase and cathepsin S mRNA and protein by atorvastatin or rosuvastatin in THP-1 macrophages treated with MSU crystals. Values presented as mean ± SEM of three independent experiments. The representative images are illustrated after three independent experiments. Abbreviation: MSU, monosodium urate.

This study demonstrated that HMG-CoA reductase inhibitors under stimulation with MSU significantly induced IL-37 expression, while down-regulating caspase-1 expression. Thus, we evaluated whether two different HMG-CoA reductase inhibitors may influence the biological activity of other proteolytic enzymes including neutrophil elastase and cathepsin S during the cleavage of precursor IL-37 into mature IL-37. We found that stimulation with MSU markedly induced two different proteases in THP-1 macrophages, but that two HMG-CoA reductase inhibitors, atorvastatin and rosuvastatin, did not have any stimulatory or inhibitory effect on mRNA and protein expression of these enzymes (Figure 4A,B). This suggests that IL-37 is activated by proteases other than caspase-1 in uric-acid-induced inflammation, and that these enzymes are not affected by HMG-CoA reductase inhibitors in the process of IL-37 activation.

## 3. Discussion

**Figure 5:**
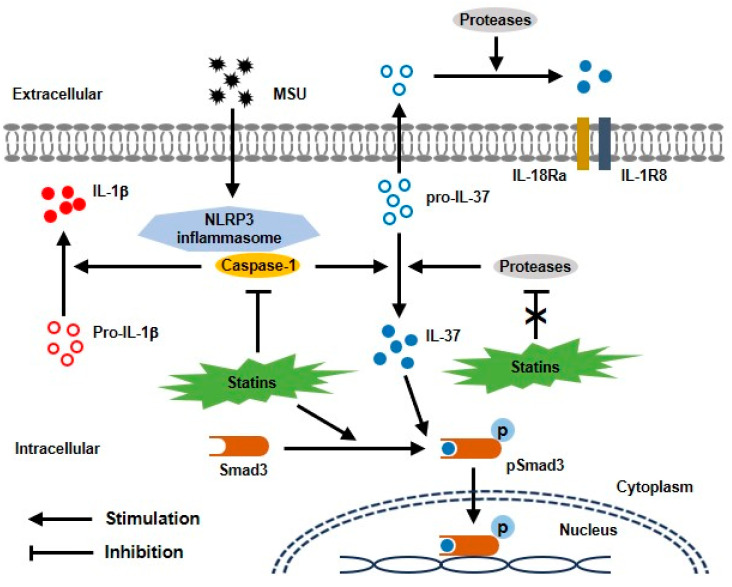
Proposed model for protective effect of statins through interaction with Smad3 and IL-37 in uric-acid-induced inflammation. MSU crystals induce activation of caspase-1 in NLRP3 inflammasome, leading to conversion of mature IL-37 from pro-IL-37. Statins block caspase-1 activation, but not other proteases in THP-1 macrophages treated with MSU crystals. In addition, statins activate phosphorylation of Smad3. Binding with activated IL-37 and Smad3 in cytoplasm is translocated into the nucleus, then stimulates transcription of anti-inflammatory molecules.

The regulation of proinflammatory cytokines has a crucial role in the inflammatory response to MSU crystals in the pathogenesis of gout [1,2]. Recent studies have implicated IL-1β as being generally considered a key proinflammatory cytokine, leading to the development of an acute gout attack. Several clinical studies have demonstrated that statin use could provide greater benefits regarding uric acid homeostasis, the risk of gout, and mortality in gout patients [25,26,27], although action mechanisms of statins for uric-acid-induced inflammatory responses have not been determined. Recently, HMG-CoA reductase inhibitors including atorvastatin, simvastatin, and mevastatin inhibited the activation of the MSU-induced NLRP3 inflammasome by the up-regulation of the peroxisome proliferator-activated receptor-γ (PPAR-γ), resulting in anti-inflammatory therapeutic action in gout [23]. THP-1 cells pre-treated with pitavastatin led to a reduction in MSU-stimulated IL-1β production and secretion through blocking caspase-1 activation [24]. To investigate the anti-inflammatory mechanism of statins in MSU-induced inflammation, we found that HMG-CoA reductase inhibitors including atorvastatin and rosuvastatin potently suppressed the release of IL-1β through up-regulation of the IL-37/Smad3 complex in THP-1 macrophages (Figure 5). 

Growing evidence has effectively demonstrated that IL-37 is implicated in the pathogenesis of chronic autoimmune and inflammatory rheumatic diseases including RA, SLE, OA, and AS [11,12,13,14,15]. Results from these clinical studies have consistently showed that the IL-37 level in the serum or PBMC was significantly higher than in healthy controls. IL-37 level was found to be closely linked with disease activity markers such as the disease activity score of 28 joints (DAS28) for RA, SLE disease activity index (SLEDAI) score and autoantibodies for SLE, visual analog scale (VAS) for OA, and Bath Ankylosing Spondylitis Disease Activity Index (BASDAI) for AS. The main mechanism of action of IL-37 is related to reducing proinflammatory cytokines and inflammatory cells including tumor necrosis factor-α (TNF-α), IL-1β, IL-6, IL-17, IL-23, and Th17 cells or increasing anti-inflammatory cytokines such as IL-10 through modulating inflammatory signal pathways. In addition, patients with gout, especially those with an active gouty attack, had significantly higher IL-37 levels in the serum, PBMC, and synovial tissue, compared to healthy controls [16,17,18]. These studies verified that the IL-37 level was significantly associated with ESR, CRP, and the presence of tophi, which implicated much higher levels of this cytokine in active gout than in inactive gout. These results are consistent with the finding of this study that the serum IL-37 level in gout was significantly higher than in controls. We also found the serum IL-37 level to be closely associated with the serum uric acid level but not with ESR and CRP in gout. In addition, the IL-37 level in human synoviocytes or synovial tissue in gout was significantly higher than that in normal controls [18,19]. In contrast, Ding et al. identified no correlation between IL-37 level and serum uric acid [17]. It has been well recognized that the main mechanism of IL-37 for limiting the inflammatory response caused by uric acid in gout is the alleviation of the release of proinflammatory cytokines and chemokines [16,17,18]. In the DNA sequencing of IL-37 using molecular inversion probe resequencing in gout patients, detection of p.(N182S)(rs752113534) carrier status was identified as a potent genetic variant favoring the risk of gout [28]. Recently, Zhao et al. demonstrated a novel opinion that IL-37 enhanced the shaping of macrophages with non-inflammatory phagocytic activity [19]. Thus, IL-37 is considered a potential biomarker for disease activity and severity and as a pathogenic target molecule in inflammatory diseases. 

Considering the biological activity of intracellular IL-37, diverse inflammatory stimuli and cytokines lead to the production of precursor IL-37 and activation of caspase-1, which in turn, trigger the cleavage of pro-IL-37 into mature IL-37. IL-37, as a transcription factor, binds to phosphorylated activated Smad3 [7,8,9,10]. The IL-37/Smad3 complex generated in the cytoplasm translocates to the nucleus to regulate gene transcription, reducing the secretion of proinflammatory cytokines and chemokines. It is considered that the binding to IL-37 and phosphorylation of Smad3 are essential in IL-37 activation. Some debates have demonstrated that HMG-CoA reductase inhibitors could regulate Smad3 phosphorylation in the TGF-β/Smad signal pathway in various cellular conditions. Accumulation of IL-37 and Smad3 in the macrophage-derived foam cells in atherosclerotic plaques was significantly inhibited by atorvastatin treatment [29]. This supports the regulation of biological activity of IL-37 by blocking Smad3 phosphorylation by statin treatment. However, IL-37 was found to be responsible for the anti-allergic contact dermatitis by regulation of Smad3 participation [30]. In addition, Liu et al. found that IL-37 potently limited the uric-acid-induced innate inflammatory response through augmentation of Smad3 and IL-1R8 expression, blocking activation of the NLRP3 inflammasome and activating SOCS in human synovial cells and THP-1 cells [18]. Consistently, we also found that two HMG-CoA reductase inhibitors, atorvastatin and rosuvastatin, activated Smad3 phosphorylation. This suggests that atorvastatin and rosuvastatin might contribute to greater translocation of the cytoplasm to the nucleus of the IL-37/Smad3 complex. 

Regarding the effect of anti-inflammatory drugs on IL-37 level, IL-37 expression together with TNF-α, IL-6, and IL-17A during the use of disease-modifying anti-rheumatic drugs (DMARDs), especially drug responders, in RA, was significantly decreased rather than before the initiation of DMARDs [11]. Similarly, mRNA expression of IL-37 and the plasma IL-37 level along with those of other inflammatory cytokines including interferon-γ and IL-6 were reduced after steroid (1 mg/kg) treatment, compared to pre-treatment [13]. It is presumed that treatment with DMARDs and a steroid suppressed the production and release of proinflammatory cytokines that can stimulate IL-37. Overexpression of IL-37 using transfection with pcDNA3.1-IL-37b or recombinant human IL-37 treatment under stimulation with MSU crystals inhibited the production of multiple inflammatory cytokines and chemokines including IL-1β, IL-6, IL-8, CCL2, and TNF-α, suggesting that augmentation of IL-37 expression effectively controls excessive gouty inflammation [16,18]. For the up-regulation of IL-37 level that plays a crucial role in limiting a diverse inflammatory response, some anti-inflammatory medicines including *Tripterygium wilfordii* Glycosides, chloroquine, and rapamycin induce IL-37 expression. These pharmaceuticals have been verified in in vitro and in vivo studies through the up-regulation of ERK1/2, p38 MAPK, and NF-κB/AP-1 signal transduction [31,32]. Consistently, we found that atorvastatin and rosuvastatin significantly induced IL-37 mRNA and protein expression in THP-1 cells treated with MSU crystals. Based on these observations, IL-37 is considered an effective therapeutic agent for inflammatory rheumatic disorders.

Several proteases including caspase-1, caspase-8, neutrophil elastase, granzyme B, cathepsin S, and cathepsin G are involved in proteolytic processing of the IL-1 family cytokines [33]. In the biological activity of IL-37, caspase-1 is primarily involved in the cleavage from pro-IL-37 to mature IL-37 and in the nuclear translocation of IL-37 [7,8]. In addition to caspase-1, IL-37 could be cleaved by additional proteases including neutrophil elastase and cathepsin S [25,34]. Active caspase-1 is well recognized to cleave pro-IL-1β to produce mature IL-1β in the process of NLRP3 inflammasome activation, which is considered a pathogenic mechanism in the pathogenesis of gout [33]. In this study to determine the role of statins in regulating IL-37 expression in the MSU-induced inflammatory response, we found that statins inhibited the activation of caspase-1. In other words, statins can be expected to block the activation of IL-1β and IL-37, but the processing of IL-37 activation was not limited. This suggests that IL-37 activation is affected by proteases other than caspase-1. In this study, we found that neutrophil elastase and cathepsin S were not inhibited by the two statins. Based on our results, it is presumed that IL-37 activation by statins is related to the activation of Smad3 phosphorylation, independent of caspase-1.

HMG-CoA reductase inhibitors are classified into two categories, namely hydrophilic statins (rosuvastatin and pravastatin) and lipophilic statins (simvastatin, atorvastatin, lovastatin, and pitavastatin), based on their water- or lipid solubility, respectively [35]. The possible superiority of either lipophilic or hydrophilic statins considering their anti-inflammatory potency has not been determined. Chang et al. demonstrated that articular chondrocytes treated with simvastatin or pravastatin with hyaluronic acid showed the down-regulated release of proinflammatory cytokines and inflammatory mediators, while hydrophilic statins resulted in better overcome [36]. Regarding the potential risk of statins and incident RA, long-term lipophilic statin use (>365 days) led to a lower possibility of the development of RA [37]. But, there was no association between hydrophilic statins or short-term use and the risk of RA. Consistently, lipophilic statins showed an anti-inflammatory action on MSU-induced NLRP3 inflammasome activation [23]. Atorvastatin and rosuvastatin used in this study equally suppressed MSU-induced inflammation through the augmentation of IL-37 production.

There are some limitations to understanding the results in this study. First, this was an in vitro studies using only THP-1 macrophages without the use of any kind of animal models or other experimental cells and it was limited in fully elucidating the pathogenic mechanism of HMG-CoA reductase inhibitors in uric-acid-induced inflammation. THP-1, an immortalized monocyte-like cell line, has been used as a model for primary human monocytes ex vivo in the research of diseases. However, there are some differences in biological functions between THP-1 cells and primary human monocytes. Therefore, data using THP-1 macrophages should be verified in studies using primary monocytes. Second, we identified the difference in serum IL-37 expression between gout and controls. But, the sample size was too small to generalize regarding the differences between the two groups. However, since other clinical studies have shown similar results [16,17], the results of this study may be considered to have some clinical significance. Third, this study did not show a long-term effect, appropriate concentration, or sustainability of the drug effect of statins on MSU-induced inflammation. Fourth, we found that Smad3 is required for showing the anti-inflammatory effect of IL-37, as demonstrated by previous studies [29,30]. Unfortunately, no other potential mechanisms for the anti-inflammatory effect of IL-37 and statins were identified. We believe that the novel and potent anti-inflammatory mechanism of action of the interaction of statins and IL-37 should be confirmed in additional studies in the future. To further verify the results of our study, additional experiments using animal models and other type of cells and clinical studies should be necessary.

## 4. Materials and Methods 

### 4.1. Study Population and Collecting Blood Samples 

This study consecutively enrolled 40 male patients who met the classification criteria for the diagnosis of gout proposed by the American College of Rheumatology/European League Against Rheumatism collaborative initiative [38]. An additional 30 healthy male subjects were recruited as controls who were age matched and without a history of gout. Inclusion criteria included subjects treated with urate-lowering drugs including febuxostat (≤80 mg/day) and allopurinol (≤300 mg/day), those over 18 years old, and those who gave informed consent. Subjects with other forms of inflammatory arthritis including RA, SLE, psoriatic arthritis, OA, and AS were excluded from both the gout and control groups by review of their medical records and by subject interview if needed. Women and subjects not receiving urate-lowering drugs were also excluded. At the time of enrollment, we collected some laboratory data such as serum uric acid (mg/dL), ESR, and CRP in patients with gout. 

### 4.2. Cell Culture 

Human monocytic cell line THP-1 cells, purchased from the Korean Cell Line Bank (Seoul, Republic of Korea), were grown in RPMI1640 (Gibco, BRL, Grand Island, NY, USA) supplemented with 10% fetal bovine serum (Hyclone, Logan, UT, USA) and 1% antibiotics (100 units/mL penicillin and 100 μg/mL streptomycin). THP-1 cells were differentiated into macrophages by 24 h of incubation with 100 nM phorbol 12-myristate 13-acetate (PMA) prior to stimulation.

### 4.3. Chemicals and Antibodies 

Atorvastatin, rosuvastatin, *p*-nitrophenylphosphate, and phorbol 12-myristate 13-acetate (PMA) were purchased from Sigma-Aldrich (St. Louis, MO, USA). Human recombinant IL-37 protein was purchased from PeproTech (Rocky Hill, NJ, USA). MSU crystals were prepared as previously described in our previous study [39].

Antibodies were purchased from the following companies: caspase-1 and Smad3 (Ser423/425) (Abcam, Cambridge, UK), IL-1β, anti-cleaved IL-1β and phospho-Smad3 (Ser423/425) (Cell Signaling, Danvers, MA, USA), IL-37 (R&D Systems, Minneapolis, MN, USA), and β-actin were obtained from Santa Cruz Biotechnology (Santa Cruz, CA, USA).

### 4.4. Enzyme-Linked Immunosorbent Assay (ELISA) 

The concentration of human IL-37 was measured by an ELISA kit according to the assay instructions (R&D Systems, Minneapolis, MN, USA). For this measurement, 96-well plates were coated with 100 μL of IL-37 captured antibody and were incubated overnight at room temperature. Plates were washed 3 times in a washing buffer (0.05% Tween 20 in phosphate buffered saline [PBS]) and blocked with 300 μL per well of 1% bovine serum albumin (BSA) in PBS for 1 h at room temperature. Serums and standards were added and then the plates were incubated for 2 h at room temperature. After washing, detection antibody was added and incubated for 2 h at room temperature. The plates were incubated with biotin-conjugated detection antibody followed by horse-radish-peroxidase-labelled streptavidin. The absorbance values of the serums were measured at 450 nm using an ELISA plate reader (BMG Lab Technologies, Offenburg, Germany).

### 4.5. Quantitative Real-Time Polymerase Chain Reaction (qRT-PCR) 

Cells were seeded on 60 mm culture dishes at a density of 8 × 10^5^ cells/dis, pre-treated with the indicated concentrations of atorvastatin and rosuvastatin for 24 h, and then stimulated with MSU (0.2 mg/mL) for 24 h. Total RNA was extracted from cells using Trizol reagent (Gibco BRL, Grand Island, NY, USA) according to the manufacturer’s instruction. A 1 μg sample of total RNAs was used for complementary DNA (cDNA) synthesis using ReverTra Ace-α-1 (Toyobo, Osaka, Japan) followed by incubation at 37 °C for 15 min, 50 °C for 5 min, and 98 °C for 5 min.

The gene expression was measured by quantitative PCR with the SYBR Green Master Mix (Toyobo, Tokyo, Japan) and a Mini Option TM Real-time PCR system (Bio-Rad, Hercules, CA, USA). For PCR amplification, 20 μL containing 2 μL cDNA, 10 µL of SYBR^®^ Green Real-time PCR Master Mix, 10 pmol/L of each primer, and 6.4 µL of distilled water were analyzed. The reaction was carried out at an initial denaturation at 95 °C for 15 min, followed by 40 cycles of 9 °C for 5 s, 58–63 °C for 30 s, and 72 °C for 15 s. 

Primer sequences were as follows: IL-37 (Forward) 5′-CGG CCC TTC ATC TTT TAT AGG-3′ and (Reverse) 5′-TTT ATC TGT CAC CCC AAC AGG-3′, NLRP3 (Forward) 5′-GAT CTT CGC TGC GAT CAA CA-3′ and (Reverse) 5′-GGG ATT CGA AAC ACG TGC ATT A-3′, Caspase-1 (Forward) 5′-GCC TGT TCC TGT GAT GTG GAG-3′ and (Reverse) 5′-TGC CCA CAG ACA TTC ATA CAG TTT C-3′, IL-1β (Forward) 5′-CCA CAG ACC TTC CAG GAG AAT-3′ and (Reverse) 5′-GTG CAC ATA AGC CTC GTT ATC C-3′, Elastase (Forward) 5′-CCC TCA CGA GAG TGC AGA CGT T-3′ and (Reverse) 5′-CGT AAA CTT CTT GCT CAA CGA CAT C-3′, cathepsin S (Forward) 5′-GCC TGT GCC TAT CAC CTC TTA T-3′ and (Reverse) 5′-CCT TCT CTG TCT GTC TCC TGG T-3′, and β-actin (Forward) 5′-CCT GAC TGA CTA CCT CAT GAA GG-3′ and (Reverse) 5′-CGT AGC ACA GCT TCT CCT TA-3′. All reactions were run in triplicate, and the relative expression of each gene was analyzed using the 2^−ΔΔCt^ method.

### 4.6. Transfection of siRNA

Transfection was performed using the Lipofectamine RNAiMAX transfection reagent (Invitrogen, Waltham, MA, USA) following the manufacturer’s protocol. Cells were seeded at 4 × 10^5^ cells/well in a 24-well plate and transiently transfected with human IL-1β or Smad3 siRNA and negative-control (NC) siRNA (Invitrogen, Waltham, MA, USA).

Briefly, siRNA was diluted in Opti-mem media (Life Technologies, Darmstadt, Germany) and mixed with 1 μL Lipofectamine RNAiMAX transfection reagent diluted in Opti-MEM media and incubated for 10 min at room temperature. Transfection complexes were added for 48 h in 24-well plates. Next, cells were pre-treated with statins for 24 h, stimulated with MSU (0.2 mg/mL) for 24 h, and prepared for subsequent experiments.

### 4.7. Western Blotting 

Cells (2 × 10^6^) were seeded on 100 mm culture dishes, pre-treated with the indicated concentrations of atorvastatin and rosuvastatin for 24 h, and stimulated with MSU (0.2 mg/mL) for 24 h. Total proteins were lysed in lysis buffer (Bio-Rad, Hercules, CA, USA) supplemented with protease inhibitor (Roche, Diagnostics, Mannheim, Germany) for 15 min on ice. The cell lysates were clarified by centrifugation at 15,000 rpm for 10 min at 4 °C, and protein concentrations were determined by a PierceTM BCA Protein Assay Kit (Thermo Fisher, Waltham, MA, USA).

Cytoplasmic and nuclear proteins were extracted using the NE-PER™ Nuclear and Cytoplasmic Extraction Reagents (Thermo Fisher, Waltham, MA, USA) according to the manufacturer’s instructions. Equal amounts of total proteins were separated on 10–12% SDS-PAGE and transferred to nitrocellulose membranes (Bio-Rad, Hercules, CA, USA) by electrophoresis. 

The membranes were blocked in 5% BSA (BD Bioscience, San Francisco, CA, USA) and probed with appropriate dilutions of primary antibodies followed by horseradish peroxidase (HRP)-conjugated secondary antibodies. Immunoreactive protein detection was performed with the ECL chemiluminescence kit (Thermo Fisher, Waltham, MA, USA). Densitometry was analyzed and quantified with Image Lab Software version 6.0 (Bio-Rad, Hercules, CA, USA).

### 4.8. Statistical Analysis

Data are described as the mean ± standard error of the mean. Statistical tests performed in this study were analyzed using nonparametric analysis according to testing for normality of the distribution using the Kolmogorov–Smirnov test. The difference in serum IL-37 levels between gout patients and controls was evaluated by the nonparametric Mann–Whiney U test. Correlation of IL-37 level with serum uric acid, ESR, and CRP in patients with gout was assessed by linear regression analysis. The statistical differences for target genes of caspase-1, IL-1β, and IL-37 mRNA, and protein expression between cells treated with each statin or control were measured by the Mann–Whitney U test. Statistical significance was considered at a *p*-value of less than 0.05. The statistical analyses and generation of plots illustrated in figures were performed by GraphPad Prism version 5.04 (San Diego, CA, USA).

## 5. Conclusions

This study found that two different HMG-CoA reductase inhibitors, atorvastatin and rosuvastatin, have anti-inflammatory effects through the augmentation of IL-37 expression due to increased phosphorylation of Smad3 in MSU-induced inflammation in THP-1 macrophages, together with the suppression of IL-1β production through blocking caspase-1 activation. HMG-CoA reductase inhibitors are suggested as being responsible for the potent negative regulation of uric-acid-induced inflammation. The clinical applicability of statins may be beneficial in the treatment of various diseases by intervening in pathogenic mechanisms [40]. IL-37 is considered an anti-inflammatory cytokine to suppress uric-acid-induced inflammation. Our finding provides evidence that recombinant IL-37 treatment or pharmacological agents that amplify the IL-37 expression might be considered as a therapeutic strategy to control acute uric-acid-induced inflammation. 

## Data Availability

The data underlying this article will be shared on reasonable request to the corresponding author.
